# Orsay virus infection increases *Caenorhabditis elegans* resistance to heat-shock

**DOI:** 10.1098/rsbl.2024.0278

**Published:** 2024-08-14

**Authors:** Victoria G. Castiglioni, Santiago F. Elena

**Affiliations:** ^1^ Instituto de Biología Integrativa de Sistemas (I2SysBio), CSIC-Universitat de València, Valencia 46980, Spain; ^2^ Santa Fe Institute, Santa Fe, NM, USA

**Keywords:** argonautes, host–virus interaction, mutualism, stress response, virus ecology and evolution

## Abstract

The heat-shock response plays a key role in the immune defence against viruses across various organisms. Studies on model organisms have shown that inducing this response prior to viral exposure enhances host resistance to infections, while deficient responses make individuals more susceptible. Moreover, viruses rely on components of the heat-shock response for their own stability and viral infections improve thermal tolerance in plants, giving infected individuals an advantage in extreme conditions, which aids the virus in replication and transmission. Here, we examine the interaction between the nematode *Caenorhabditis elegans* and its natural pathogen the Orsay virus (OrV) under heat stress. We found that OrV infection leads to differential expression of heat-stress-related genes, and infected populations show increased resistance to heat-shock. This resistance correlates with increased expression of argonautes *alg-1* and *alg-2*, which are crucial for survival after heat-shock and for OrV replication. Overall, our study suggests an environmental-dependent mutualistic relationship between the nematode and OrV, potentially expanding the animal’s ecological niche and providing the virus with extra opportunities for replication and adaptation to extreme conditions.

## 1. Introduction

Viruses have traditionally been viewed as pathogens that harm their hosts while ensuring their own survival. However, recent studies are delineating a different picture, revealing that viruses are ubiquitous symbionts and not always linked to diseases. Additionally, some viruses have been described to be advantageous to the host by inducing tolerance to different abiotic stresses [1]. This has led to the understanding that the outcome of an infection hinges on environmental factors, with instances in which virulence is increased under abiotic stresses and situations in which more severe stresses result in reductions of pathogen’s prevalence [2].

In the context of global warming, an environmental factor of particular relevance to better forecast the evolution and emergence of infectious diseases is thermal stress. Thermal stresses activate the heat-shock response (HSR), a cellular mechanism that prevents proteotoxicity, and has diverse interactions with viral infections. First, the HSR forms part of the antiviral immune response in a multitude of organisms, from plants to insects and mammals [3–5]. Induction of a HSR in mice prior to exposure to a virus increases the host resistance to the viral pathogen [6], while HSR transcription factor deficient adult flies are hypersensitive to viral infection [7]. Second, some viruses require components of the HSR, such as Hsp90, for successfully completing their infectious cycle [8,9]. Third, viral infection has been reported to enhance thermal tolerance in some plants, conferring infected individuals an advantage over extreme conditions and enabling viruses to continue replicating and transmitting within the population [1].

In *Caenorhabditis elegans*, induction of the HSR has been shown to increase resistance to bacterial infection [10], and infection with the intracellular microsporidium *Nematocidia parisii* induces upregulation of gene sets also upregulated by heat-shock treatments [11]. Moreover, induction of the HSR increases resistance to infection by Orsay virus (OrV) [12].

OrV has a positive-sense, bi-segmented RNA genome of ~6.3 kb which encodes four proteins: the RNA-dependent RNA polymerase (RdRP), the viral capsid protein (CP), the δ protein and a protein fusion of CP-δ [13,14]. OrV replicates in the intestinal epithelia of *C. elegans,* and infection results in enlarged intestinal lumens and subcellular structural changes but does not affect animal life span and has a small effect on brood size [13,14]. Some of the genes involved in the immune response against OrV are also involved in tolerance to abiotic stressors [15–17], and OrV infection alters the transcriptomic response of *C. elegans* to heat-shock [12].

In this study, we sought to further characterize the three-way interaction between *C. elegans*, its natural pathogen OrV and an environmental heat-shock stress. To do so, we first looked into the transcriptional response elicited in OrV infected animals focusing on genes related to HSR, we then analysed the resistance of infected populations to heat stress and finally we characterized the expression of argonaute genes *alg-1* and *alg-2* in infected and control populations upon heat-shock. These two argonautes have been already described as involved in animal’s survival to heat-shock [18]. Moreover, *alg-1* is a proviral factor necessary for OrV replication [19]. Altogether, we describe links between the HSR and the transcriptional response to OrV and a protective effect of virus infection to heat-shock, increasing the resistance of *C. elegans* to a mildly lethal heat-shock.

## 2. Methods

### (a) Overlap of heat-stress-related Gene Ontology terms and Orsay virus differentially expressed genes

The differentially expressed genes (DEGs) upon OrV infection in wild-type animals were obtained from [20]. The genes belonging to the Gene Ontology (GO) term categories (i) cellular response to unfolded proteins, (ii) heat-shock binding proteins and (iii) response to heat was extracted from WormBase Ontology Browser [21]. Only genes annotated as directly involved in each category were used. Genes that were differentially expressed upon OrV infection and belonged to any of these three GO term categories were selected.

### (b) Strain maintenance

Nematodes were maintained at 20°C on Nematode Growth Medium (NGM) plates seeded with *Escherichia coli* OP50 under standard conditions [22,23]. ERT54 (*jyIs8 [pals−5p::GFP; myo−2p::mCherry]X* in an N2 Bristol background [11]) served as wild-type. JU2624, (*mjIs228[myo−2p::mCherry::unc54; lys−3p::eGFP::tbb−2]* in a JU1580 background) was the natural isolate from which OrV was first identified. Both strains contain fluorescent reporters that get activated upon infection, which were used to discern between infected and non-infected animals. The strains were generously provided by Prof. M. A. Félix [13].

### (c) Orsay virus stock preparation and quantification

JU2624 animals were inoculated with OrV isolate JUv1580 (a gift from Prof. M. A. Félix [13]), allowed to grow for 5 days and then resuspended in M9 buffer (0.22 M KH_2_PO_4_, 0.42 M Na_2_HPO_4_, 0.85 M NaCl, 0.001 M MgSO_4_), left to stand for 15 min at room temperature, vortexed and centrifuged for 2 min at 400*g*. The supernatant was centrifuged twice at 21 000*g* for 5 min and then passed through a 0.2 μm filter. RNA of the resulting viral stock was extracted using the Viral RNA Isolation kit (NZYTech). The concentration of viral RNA was determined by RT-qPCR and normalized using a standard curve. Primers used can be found in table 1.

**Table 1.** Sets of primers used for different purposes.

primer name	primer sequence (5′–3′)	application	
VG9_RNA1_qPCR_1_F	TTCCTGTCCAGGCAGTTCTA	RT-qPCR OrV	
VG10_RNA1_qPCR_1_R	GATGGATCTTGGCAAGCAGA	RT-qPCR OrV	
VG15_RNA2_qPCR_2_F	ACGAAGCAGTAGCCGTTAAG	RT-qPCR OrV	
VG16_RNA2_qPCR_2_R	GAGAACATCCTTCTCTGCGG	RT-qPCR OrV	
VG5_OrV_RNA1_3′_F	TAATACGACTCACTATAGGTTCCTGTCCAGGCAGTTCT	standard curve	
VG6_OrV_RNA1_3′_R	GGACCTCTCCTGGGTATGTG	standard curve	
VG7_OrV_RNA2_3′_F	TAATACGACTCACTATAGGCCTGTCAGAGTTGAGAACA	standard curve	
VG8_OrV_RNA2_3′_R	ATAGCCGGGTATGGATAGCG	standard curve	
cdc42_1F	AGCCATTCTGGCCGCTCTCG	RT-qPCR *cdc-42*	
cdc42_1R	GCAACCGCTTCTCGTTTGGC	RT-qPCR *cdc-42*	

For the standard curve, cDNA of OrV was obtained using Accuscript High Fidelity Reverse Transcriptase (Agilent) and reverse primers at the 3′ end of the virus (table 1 for primers). Approximately 1000 bp of the 3′ end of RNA1 and RNA2 were amplified using forward primers containing 20 bp coding the T7 promoter and DreamTaq DNA Polymerase (ThermoFisher). The PCR products were gel-purified using MSB Spin PCRapace (Invitek Molecular) and an *in vitro* transcription was performed using T7 Polymerase (Merck). The remaining DNA was then degraded using DNase I (Life Technologies). RNA concentration was determined by NanoDrop (ThermoFisher), and the number of molecules per microlitre was determined using the online tool EndMemo RNA Copy Number Calculator (https://endmemo.com/bio/dnacopynum.php).

### (d) Nematode synchronization and Orsay virus inoculation

In order to obtain synchronized populations of wild-type and JU2624 animals, plates with eggs were carefully washed with M9 buffer to remove larvae and adults but leaving the eggs behind. Plates were washed again using M9 buffer after 1 h to collect larvae hatched within that time span. Synchronized nematode populations were inoculated with 4.92 × 10^8^ copies of OrV—a concentration that leads to activation of the *pals-5p::GFP* reporter [11] in ~50% of the animals—by pipetting the viral stock on top of the bacterial lawn containing the animals. Twenty-four hours post-inoculation (hpi) *pals-5p::GFP* or *lys-3p::eGFP* negative animals were manually removed from infected plates, and negative control plates were checked to confirm the absence of reporter activation.

### (e) Heat-shock

Synchronized populations were shifted from a 20°C incubator to a 37°C water bath at 48 hpi for 2 h. This heat-shock treatment results in ~50% mortality of control animals, and hereafter we will refer to it as semi-lethal. Plates were sealed with parafilm and placed bottom down in order to ensure a quick temperature shift. After the heat-shock, they were returned to 20°C.

### (f) Mortality assay

Twenty-four hours post-heat-shock (hphs) mortality within the heat-shocked population was quantified. Animals were considered dead when they did not react to touch and had stopped pharyngeal pumping.

Mortality was evaluated in four and eight independent full blocks for JU2624 and wild-type populations, respectively. The number of plaques (biological replicates) within each experimental condition within blocks varied between 2 and 13 (median 3). Mean and standard deviation of mortality were estimated for each infection condition, nematode strain and experimental block. Data were analysed using a meta-analysis approach for continuous data, with infection status considered as a fixed factor, using Cohen’s *d* (±1) as a normalized estimation of the effect size, and restricted maximum-likelihood for parameter’s estimation. These analyses were done using SPSS v. 29.0.1.1 (IBM Corp).

### (g) Quantification of *alg-1* and *alg-2* expressions

To quantify expression levels of *alg-1* and *alg-2,* wild-type control and infected animals were used. Nematodes were heat-shocked as described above, and samples were taken before heat-shock, right after the heat-shock and 2 and 8 hphs. Three biological replicates, each consisting of a pool of 80 animals, were used for each time-point and condition. Animals were collected with PBS 0.05% Tween and washed three times before freezing in liquid N_2_. RNA was extracted using Trizol as previously described [18]. RT-qPCRs were performed using Power SYBR Green PCR Master Mix (Applied Biosystems) on an ABI StepOne Plus Real-time PCR System (Applied Biosystems). Ten nanograms of total RNA were loaded and samples were normalized to expression of *cdc-42*. Primers used can be found in table 1. Relative expression levels were computed using the ΔΔ*C*_T_ method [24].

Fold-change values for each mutant genotype were independently fitted to a generalized linear model with a normal distribution and identity function with infection status and hpsh incorporated as orthogonal random factors. The interaction between the two factors is reported in the text as an evaluation of the differences in temporal expression dynamics. These analyses were also done with SPSS v. 29.0.1.1.

## 3. Results

**Figure 1.**
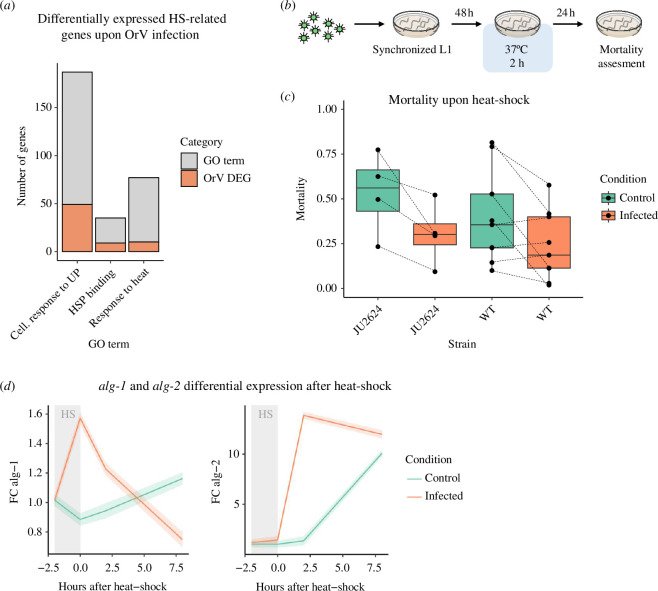
Interplay between OrV infection and HSRs in *C. elegans*. Significant enrichment of genes related to HSRs that are associated with viral infection (*a*). Schematic representation of the experiments performed. Four independent blocks were performed with JU2624 and eight with the wild-type (*b*). Effect of OrV infection in the mortality-induced heat-shock. *P* values were obtained from the meta-analysis of experimental blocks as described in the Methods section. Grey lines connect mortality values of the same experimental block (*c*). Analysis of the induction of two argonaute genes, *alg-1* and *alg-2*, upon heat-shock in control and OrV-infected animals. FC: fold-change in gene expression measured by RT-qPCR. Error bands are denoted by shading above and below the solid line and represent ±1 standard errors of the estimated marginal means (*d*).

In this study, we examined the relationship between the nematode *C. elegans* and its natural parasite, OrV [13], with and without heat stress. We first looked into the transcriptional response elicited in infected animals, focusing on genes related to HSR. For this purpose, we screened the DEGs in wild-type animals upon OrV infection [20] for genes in the GO categories of ceJU2624 animals carry a mutation llular response to unfolded proteins, heat-shock binding proteins and response to heat (§2(a)). The three categories were overrepresented (Fisher’s exact test) among the significant DEGs upon OrV infection (figure 1*a*): 26.3% (*p* < 0.001), 26.47% (*p* < 0.001) and 13.2% (*p* = 0.012), respectively. Among these DEGs, 60.7% were upregulated, suggesting that the transcriptional response induced by OrV has a strong overlap with the response elicited by a heat-shock.

We then examined the ability of infected populations to withstand a semi-lethal heat-shock (figure 1*b*). Infected populations of JU2624, whose genetic background corresponds to the natural isolate in which OrV was first identified, had a significant decrease in mortality upon heat-shock (figure 1*c*; Cohen’s *d* = −1.974 ± 0.616, *p* = 0.001) suggesting that OrV infection is able to confer thermal tolerance to *C. elegans*. In contrast, the decrease observed in wild-type animals was moderate but non-significant (figure 1*c*; *d* = −0.632 ± 0.334, *p* = 0.058). This difference among host genetic backgrounds suggests a potential role of previous host–virus coevolution in the magnitude of the protection conferred by infection. Nonetheless, when comparing control and infected populations irrespective of their genetic background, the observed infection-associated reduction in mortality due to heat-shock was largely significant (*d* = −0.936 ± 0.293, *p* = 0.001).

Next, in order to identify some of the players involved in this response, we looked at the expression of the argonautes *alg-1* and *alg-2*, whose products have been shown to be involved in survival after heat-shock due to their role in microRNA biogenesis [18]. In addition, *alg-1* has also been proven to be a necessary factor for OrV replication [19]. Upon heat-shock, the expression of these two argonaute genes increased significantly in infected versus control wild-type populations, showing quite different temporal dynamics (figure 1*d*). In the case of *alg-1* (*χ* ^2^ = 81.856, 3 d.f., *p* < 0.001), infection caused a sharp increase in its expression levels right after heat-shock, which decreased at 8 hphs, at which point control animals displayed significantly higher expression levels of *alg-1*. In turn, infection caused a sharp increase in the expression levels of *alg-2* at 2 hphs and its levels remained high until 8 hphs (*χ* ^2^ = 117.538, 3 d.f., *p* < 0.001), at which point the expression of *alg-2* also increased in control animals. These short but sharp increases in *alg-1* and *alg-2* levels may be critical in the increased thermal tolerance caused by OrV infection, although more genetic and functional studies would be necessary to fully elucidate the roles of these proteins in thermal tolerance and its interplay with viral infections.

## 4. Discussion

Infection of *C. elegans* with OrV results in a mild phenotype, with a small reduction in progeny size but no effect on lifespan. This makes it a convenient pathosystem to study the interactions between host, virus and abiotic stress, particularly in regards to potential mutualistic relationships. Here we examined the interplay between *C. elegans*, OrV and the HSR. We showed that infection of *C. elegans* by OrV causes differential gene expression of many genes related to HSR. Moreover, we observed that OrV infection confers protection against a semi-lethal heat-shock in a host genotype-dependent manner. This protection may be, at least partially, explained by the upregulation of the argonautes *alg-1* and *alg-2*, which are required for survival after heat-shock and OrV replication.

We described that the extent of the mutualistic relationship depends on the previous coevolutionary history between both partners. In this sense, the OrV-induced protection against heat-shock was larger in animals of the JU2624 genotype, from which OrV was first isolated, than in animals of the ERT54 wild-type genotype without previous history of coevolution with this particular isolate of the virus. Notably, the protective effect that heat-shock confers to infection may also depend on the genetic background, with animals of the natural isolate in which OrV was first identified having more dramatic reductions in viral load after heat-shock than wild-type animals [12]. JU2624 animals carry a mutation in *drh-1(niDf250*), which makes them more susceptible to viral infection [14]. This allele is particularly prevalent in French populations of *C. elegans* [14], from where the only two described OrV strains come from [13,25]. In The Netherlands, *C. elegans* infecting viruses are rare and may only be present in 1.5% of the natural populations [26]. Natural isolates from these two regions, as well as from elsewhere, might have adapted to their local temperature variations to improve their reproductive rate [27]. Therefore, as the effort continues to describe new OrV strains across the world, it will be interesting to confirm whether the protective effect against heat-shock conferred by OrV is generalized across *C. elegans* isolates and across viral strains, and whether the heat resistance provided by OrV was a factor in the coevolution of OrV and the natural isolates in which different OrV strains were first identified. The particular niche in which a virus has evolved can dramatically affect its relationship with its host. The mutualistic relationship between hosts and viruses may have broad ecological consequences for both partners. On the one side, the infected host may expand its niche and afforded increasing chances to adapt to even more extreme scenarios. On the other side, by keeping its host alive, within-host replication opportunities for the virus are enhanced, promoting transmission. Host–virus mutualistic relationships have been pervasively described for plant viruses [1]. Indeed, plant virus experimental evolution under strong drought resulted in a transition from parasitism to mutualism [28]. Our study shows that mutualistic interactions can also take place between an animal virus and its host under harsh temperature conditions. This mutualistic relationship could also have consequences on *C. elegans* adaptation to extreme conditions. More studies would need to be done in order to (i) understand if this mutualistic relationship affects the nematodes’ ecological niche breadth, (ii) also takes place with other animal viruses and (iii) investigate whether the same mutualistic relationship also applies to different stresses.

## Data Availability

Excel files containing all raw data as well as description of methods are available from Dryad [29].
